# A combination of coenzyme Q10, feverfew and magnesium for migraine prophylaxis: a prospective observational study

**DOI:** 10.1186/s12906-017-1933-7

**Published:** 2017-08-30

**Authors:** Angèle Guilbot, Marie Bangratz, Samira Ait Abdellah, Christian Lucas

**Affiliations:** 1 Pileje Laboratoire, 37 quai de Grenelle, 75015, 15 Paris cedex, France; 2 Centre d’Evaluation et de Traitement de la Douleur, service de neurochirurgie, Hôpital Salengro, CHRU de Lille, 59037 Lille Cedex, France

**Keywords:** Migraine, Feverfew, Magnesium, Coenzyme Q10

## Abstract

**Background:**

Feverfew (*Tanacetum parthenium L.*), magnesium and coenzyme Q10 are frequently used for migraine prophylaxis. Supplementation with a fixed combination of these three agents (Antemig®, PiLeJe) was investigated in an observational study.

**Methods:**

Adult patients suffering from migraine according to the criteria of the International Headache Society were enrolled by general practitioners (≥2 migraine attacks during previous month; exclusion of chronic migraine and medication overuse) and after a one-month baseline phase, supplemented with one tablet of 100 mg feverfew, 100 mg coenzyme Q10 and 112.5 mg magnesium per day for 3 months.

**Results:**

Supplementation significantly reduced the number of days with migraine headache during third month of supplementation compared to baseline phase (1.3 days ±1.5 versus 4.9 days ±2.6, *p* < 0.0001; *n* = 68 intention to treat; primary criterion). The decrease was progressive over the period of supplementation and significant from first month (1st month: −2.5 days ±3.1, *p* < 0.0001; 2nd month: −3 days ±2.8, *p* < 0.0001). The proportion of patients with a reduction of at least 50% in the number of days with migraine headache was 75% (51/68) after 3 months, with a progressive increase over the period of supplementation (63.2% [43/68] after 1 month and 70.6% [48/68] after 2 months). The proportion of patients with anxiety and depressive symptoms (Hospital Anxiety and Depression Scale) decreased between baseline phase and third month of supplementation from 61.9% (39/63 patients with information available) to 35% (21/60) for depression and from 52.4% (33/63) to 30% (18/60) for anxiety. An improvement of quality of life (Qualité de Vie et Migraine questionnaire) was also observed. The combination was well tolerated.

**Conclusions:**

Results suggest that the proprietary supplement containing feverfew, coenzyme Q10 and magnesium assessed could be beneficial and safe for the prevention of migraine in adult patients and merits further study.

**Trial registration:**

ClinicalTrials.gov: NCT02901756, retrospectively registered on August 24, 2016.

## Background

Migraine is a common neurological disorder characterized by severe episodic headaches, which are frequently incapacitating. US government statistics indicate that migraine affects around one in seven Americans annually [1]. European data showed the mean prevalence of migraine among 170,000 adults to be 14.7% (8% in men and 17.6% in women) [2]; in France, prevalence was reported to be between 12 to 21% in the Framig III study [3].

Migraine treatment traditionally includes acute therapy for aborting migraine attacks and prophylactic treatment for reducing the frequency, duration and severity of attacks. According to the European Federation of Neurological Societies (EFNS) guideline, prophylactic treatment should be considered when the quality of life is severely impaired, when two or more attacks occur per month, when migraine attacks do not respond to acute therapy or in case of frequent, very long, or uncomfortable auras [4]. Despite the multitude of treatment options available, patients are often dissatisfied due to failure of achieving optimal control and cost of conventional drugs. In addition, some of the existing medications are associated with unacceptable adverse effects [5]. The need for effective treatments with less side effects explains, at least partly, the increasing interest toward non-pharmacologic alternatives for migraine prophylaxis, which include vitamins, minerals, and supplements along with herbal preparations.

One of the best-studied plants for migraine prophylaxis in adults is feverfew (*Tanacetum parthenium L.*). Antimigraine properties are attributed to its main active component, parthenolide, which has multiple actions including vascular smooth muscle relaxation, inhibition of serotonin release from platelets and anti-inflammatory effects [6, 7]. Efficacy of a feverfew monopreparation in migraine prevention was shown in a randomised double blind placebo-controlled clinical study [8]. However, efficacy was not observed in all studies performed with feverfew. This inconsistency in results between studies could be due to the variations in the concentration and stability of parthenolides in the extracts tested [7].

Coenzyme Q10 has been hypothesized to be useful in migraine prevention because of its important role in sustaining mitochondrial energy stores [6] and the evidence that migraine results in mitochondrial energy deficiency in the brain [9–11]. It was also reported that coenzyme Q10 counteracts endothelial dysfunction by stimulating endothelial release of nitric oxide and has anti-inflammatory effects [6]. A study conducted in 1550 children and adolescents with migraine showed that about one third of them had a coenzyme Q10 level below the reference range [12]. Supplementation of this subpopulation with coenzyme Q10 (1 to 3 mg/kg per day for three months) improved coenzyme Q10 level, headache frequency and disability. In a double blind, randomised, placebo-controlled clinical trial performed in adult patients with migraine [13], treatment with coenzyme Q10 (100 mg per os three times a day for 3 months) significantly decreased the frequency of migraine attacks.

There is a large body of evidence that points to a state of magnesium deficiency in migraine sufferers that could contribute to migraine development [6, 14, 15]. Magnesium is involved in a multitude of biological processes, some of which being linked to migraine pathogenesis (ATP production and function, glucose metabolism, control of vascular tone) [7]. In a randomised placebo-controlled study, supplementation with magnesium (600 mg per os daily for 3 months) significantly reduced the frequency of migraine attacks in adult patients [16]. Efficacy of magnesium was also reported specifically in adults with migraine without aura [17]. In children, it was shown that pre-treatment with magnesium increased the efficacy of acetaminophen and ibuprofen [18]. No beneficial effect of magnesium for the prevention of migraine was also reported [19].

The American Academy of Neurology (AAN) and American Headache Society (AHS) guideline for migraine prevention in adults gave feverfew and magnesium a Level B recommendation (probably effective and should be considered for prevention) [20] and the EFNS guideline a Level C (probable efficacy) [4]. The AAN/AHS and EFNS guidelines gave coenzyme Q10 a Level C recommendation.

Based on this information, supplementation with these three ingredients simultaneously might be of benefit for migraine sufferers. We conducted an observational study to assess a 3-month supplementation with a proprietary supplement containing feverfew, coenzyme Q10 and magnesium (Antemig®) in adults diagnosed with migraine according to the criteria of the International Headache Society; the number of days with migraine headache per month was the primary outcome.

## Methods

### Study design and ethic statement

This was a noninterventional prospective observational study performed in France between January and December 2015. The study was conducted in a context of routine practice; all the acts were practised and supplement used in a usual way without any additional or unusual procedure of diagnosis or surveillance. The study was approved by the relevant French authorities for this type of studies: the Advisory Committee on Information Processing in Material Research in the Field of Health (ethic committee) and the National Commission on Informatics and Liberty. The study was performed in accordance with the ethical standards laid down in the Declaration of Helsinki and the Strengthening the Reporting of Observational studies in Epidemiology (STROBE) guidelines. All patients gave their informed written consent prior to study participation.

### Participants and recruitment

Adult patients aged 18 to 65 years of either sex were recruited by general practitioners (GPs) who were used to recommend the dietary supplement to patients suffering from migraine: patients would have received the combination regardless of whether the study was taking place. Patients had (1) to suffer from migraine with or without aura diagnosed according to the criteria of the International Classification of Headaches Disorders-III (ICHD-III 1.1 and 1.2) [21]; (2) to suffer from migraine for more than one year; (3) to be less than 50 years old at migraine onset; and (4) to have had at least two migraine attacks during the month before recruitment. Non-inclusion criteria were: (1) migraine with aura with motor symptoms (hemiplegic migraine); (2) more than 15 migraine attacks per month; (3) abuse of painkillers defined as the use of paracetamol, aspirin and non-steroid anti-inflammatory drugs for more than 15 days per month over the last three months or the use of triptans, opioids and ergot-type medications for more than 10 days per month during the last three months; and (4) prophylactic treatment taken for less than 3 months. Patients who were taking a prophylactic treatment for more than 3 months were allowed to participate in the study because it was considered that after three months, prophylactic treatment was installed and therefore would not have any impact on the effect of supplementation tested.

### Supplementation

The dietary supplement was a combination of feverfew, coenzyme Q10 and magnesium (Antemig®, PiLeJe, France) marketed in France since 2014. One tablet contains 100 mg of feverfew, 100 mg of coenzyme Q10, 112.5 mg of magnesium and 1.4 mg of vitamin B6 (to facilitate magnesium absorption). The supplement has to be taken at the dose of one tablet every morning for three months.

### Procedure

The procedure was compliant with routine practice and the official recommendations for the management of migraine in adults [22]. On the first visit (V1), GPs collected demographic data, medical and migraine history and information on migraine attacks, associated symptoms and rescue medications via an electronic case report form. V1 was followed by a one-month observation period (baseline phase) during which migraine treatment of patients was not modified. From V1 till the end of the study, all patients kept an electronic diary in which they reported migraine characteristics (number of days with migraine headache, intensity, associated symptoms and concomitant medications). On the second visit (V2; Day 30 ± 10), eligibility of patients was verified: patients with less than 2 or more than 15 migraine attacks during baseline phase and patients unwilling to be supplemented with the combination were excluded from the study. Eligible patients were advised to start supplementation. On V3 (Day 120 ± 10), information provided in patient’s diary and compliance with supplementation were checked.

The day before V2 and V3, patients had to complete the French migraine-related quality of life questionnaire (Qualité de Vie et Migraine [QVM]) and the Hospital Anxiety and Depression Scale (HADS). The QVM contains 20 questions to evaluate psychological, functional/somatic, and social repercussions of migraine, as well as disturbances generated by treatment [23]. On the basis of the mean score obtained for each dimension, a global index is calculated and reported on a scale of 0 (best quality of life) to 100 (more altered quality of life). The HADS consists of 14 items divided into two seven-item subscales, each having a total score ranging from 0 to 21. A score ≤ 7 corresponds to “no depression or anxiety,” a score of 8–10 to “minor depression/anxiety”, a score > 10 to “moderate or severe depression/anxiety” [24].

### Evaluation criteria

The primary evaluation criterion was the number of days with migraine headache during third month of supplementation. Reduction of at least 50% in the number of days with migraine headache, associated symptoms, quality of life (QVM), anxiety/depression (HADS), safety and compliance were the secondary evaluation criteria.

### Statistical analysis

For calculation of sample size, we hypothesized a 50% decrease in the number of days with migraine per month after 3 months of supplementation assuming an average of two crises per month and a standard deviation (SD) of 1.3 before supplementation. Given a statistical power of 90% and an error probability of 0.05, the optimal number of participants was estimated to be 72.

Continuous variables are presented as mean ± SD. Categorical variables are presented as percentages. The chi-squared test was used to assess differences between categorical variables. The Shapiro-Wilk test was used to test each variable for normality. Student’s t-test or Mann-Whitney U test was used depending on the normality or non-normality of the data distribution. The McNemar test for matched samples was used to compare the evolution of associated symptoms, severity and distribution of HADS scores. In all tests, *p* values <0.05 were considered statistically significant. The principal analysis was performed on the intention to treat (ITT) population. A second analysis performed for confirmatory purposes was done in subjects who had respected the stipulated interval between visits (± 10 days) and who had received at least 75% of the scheduled study treatment (per protocol [PP] population). Safety analysis was performed on all subjects having received at least one dose of treatment. Confounding factors were sought using variables of interest and baseline characteristics. The following parameters were tested: sex, type of migraine (with or without aura), at least one medical history, at least one family history, centre, age at migraine onset, age at baseline, BMI, duration of migraine attacks without treatment, frequency of migraine attacks at baseline. A nonparametric Spearman correlation test was performed for quantitative variables and a Wilcoxon-Mann-Whitney test for qualitative variables. A multivariate Generalized Estimating Equation (GEE) model was used to test treatment effect adjusted on confounding factors with *p* < 0.20 in the univariate analysis. All statistical analyses were performed using SAS 9.4 (Cary, NC).

## Results

**Fig. 1:**
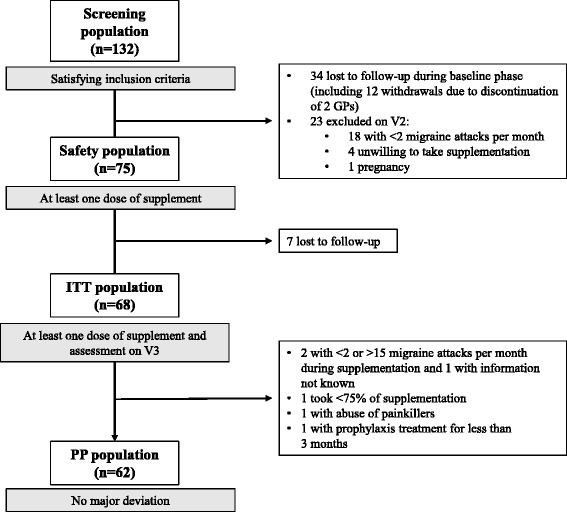
Disposition of patients and data sets

A total of 132 patients were screened by 20 GPs between January and December 2015. Thirty-four patients were lost to follow-up during the baseline phase (two GPs discontinued the study [one died] leading to the loss of 12 patients) and 23 patients were excluded on V2 (Fig. 1). Therefore, 75 patients (Safety Population) started supplementation on V2. Sixty-eight patients received at least one dose of supplement and were assessed on V3 (ITT population) and 62 subjects were included in the PP population.

### Baseline characteristics

Patients were mainly women (91.2%, 62/68 patients; ITT population; Table 1). Mean age at inclusion was 43.9 years ±11.2 and mean age at onset of migraine was 23.9 years ±10.9 (median: 20 years). A majority of patients (73.5%, 50/68) was diagnosed with migraine without aura. In patients with migraine with aura (26.5%, 18/68), aura was more commonly characterized by visual manifestations (94.4%, 17/18). More than half of patients (52.9%, 36/68) had at least one family member who was also suffering from migraine, more frequently the mother (63.9%, 23/36). Thirty-six patients (52.9%) had a medical history including 19 (27.9%) who declared to have suffered or to suffer from depression. The majority of women were premenopausal (68.9%, 42/61 women with information available); among them 12 (28.6%) declared to have menstrual migraine.

**Table 1:** Baseline characteristics

Characteristics	ITT population (*n* = 68)	PP population (*n* = 62)
Women	62 (91.2%)	56 (90.3%)
Men	6 (8.8%)	6 (9.7%)
Age	43.9 ± 11.2	43.5 ± 10.7
Migraine with aura	18 (26.5%)	14 (22.6%)
Migraine without aura	50 (73.5%)	48 (77.4%)
Age at migraine onset	23.9 ± 10.9	23.8 ± 10.5
Medical history^a^	36 (52.9%)	34 (54.8%)

^a^number (%) of patients who declared to have suffered or to suffer from at least one disease

At inclusion, prophylactic treatment for more than 3 months was taken by 13.2% of patients (9/68). Medication taken for acute treatment of migraine (97.1%, 66/68) was more often non-steroidal anti-inflammatory drugs (43.9%, 29/66). Eight patients (11.8%) continued their prophylactic treatment during supplementation: 4 patients took propranolol, 1 *Harpagophytum procumbens* and 1 vitamin B6 (missing information for 2 patients).

### Number of days with migraine headache per month

Supplementation with the combination of feverfew, coenzyme Q10 and magnesium significantly reduced the mean number of days with migraine headache per month (Table 2). During the third month of supplementation, patients declared 1.3 days ±1.5 with migraine headache compared to 4.9 days ±2.6 during baseline phase (−3.5 days ±2.9; *p* < 0.0001). The decrease was progressive over the period of supplementation and statistically significant from first month (1st month: −2.5 days ±3.1, *p* < 0.0001; 2nd month: −3 days ±2.8, *p* < 0.0001).

**Table 2:** Number of days with migraine headache per month and 50% reduction in this number

	ITT population (*n* = 68)	PP population (*n* = 62)
No. of days with migraine headache per month (mean ± SD)		
Baseline phase	4.9 ± 2.6	5.1 ± 2.5
1^st^ month	2.4 ± 3.1*	2.5 ± 3.2*
2^nd^ month	1.9 ± 2.3*	2 ± 2.4*
3^rd^ month	1.3 ± 1.5*	1.4 ± 1.6*
% of patients with ≥ 50% reduction in no. of days with migraine headache per month (% [CI 95%])		
1^st^ month	63.2% [51.8–74.7]	64.5% [52.6–76.4]
2^nd^ month	70.6% [59.8–81.4]	71.0% [59.7–82.3]
3^rd^ month	75% [64.7–85.3]	74.2% [63.3–85.1]

*p < 0.0001 versus baseline phase

*CI* confidence interval

### At least 50% reduction in number of days with migraine headache per month

The proportion of patients with a reduction of at least 50% in the number of days with migraine headache per month was 75% (51/68) after 3 months of supplementation (Table 2). The proportion progressively increased during the period of supplementation: 63.2% (43/68) after 1 month and 70.6% (48/68) after 2 months.

### Intensity of migraine headache

Before supplementation, more than half of migraine headaches were of moderate intensity (56.7%, 202/356), 25.3% (90/356) were mild and 18.0% (64/356) severe. There was no significant change in the intensity of migraine headaches during the period of supplementation (at 1, 2 and 3 months).

### Associated symptoms

Before supplementation, migraine attacks were associated with light sensitivity (93.7%, 59/63 patients with information available), noise sensitivity (93.4%, 59/63), nausea (73%, 46/63), and sensitivity to odours (58.7%, 37/63). Associated symptoms reported in less than 50% of patients were dizziness (49.2%, 31/63) and vomiting (39.7%, 25/63). Globally, associated symptoms were less frequent during supplementation. During the third month of supplementation, a significant decrease in the number of patients with sensitivity to light (−55%, *p* < 0.05) and noise (−37.5%, *p* < 0.05) was observed. Frequency of nausea was significantly reduced on the first (−45%, *p* < 0.01) and second month (−63.6%, *p* = 0.001) of supplementation.

### Anxiety and depression

At baseline, mean scores for HADS-Anxiety and HADS-Depression were 8.8 ± 3.5 and 7.8 ± 3.3, respectively. A significant decrease in the mean of both the HADS-Anxiety and HADS-Depression scores was observed after three months of supplementation (6.2 ±  3.3 [*p* < 0.0001] and 6.2 ± 3.6 [*p* = 0.0003], respectively). Using a HADS cut-off value of 8, prevalence of anxiety and depressive symptoms before supplementation was 61.9% (39/63 patients with information available) and 52.4% (33/63), respectively. After supplementation, anxiety symptoms were present in 35% (21/60) of patients and depression symptoms in 30% (18/60). The comparison of the overall distribution of classes was significant (*p* < 0.005 for HADS-anxiety and *p* < 0.05 for HADS-depression).

### Quality of life

Three months of supplementation improved the quality of life of patients as assessed by the QVM score. The global QVM score was significantly decreased from 53.1 ± 15.9 (*n* = 63) at baseline to 39.5 ± 14.4 (*n* = 60) at 3 months (*p* < 0.0001). A significant improvement was also observed for the scores of the four dimensions assessed with the questionnaire (psychological, functional/somatic, social and therapeutic).

All significant results reported for the ITT population were also observed in the PP population.

The multivariate analyses confirmed the results presented above. The effect of treatment was significantly associated with a reduction in the number and frequency of migraine attacks, HAD-depression, HAD-anxiety and QVM scores (*p* < 0.0001).

### Compliance and safety

In the ITT population, information on compliance was available for 66 patients: 65 declared to have taken the dietary supplement, 56 without interruption and 9 with missed days (number of days without supplementation: 27.6 ± 27.2 [*n* = 8]; median: 14 [10;50]).

Supplementation was well tolerated. Four patients out of 75 (Safety Population) experimented one adverse event each: two “digestive troubles”, one “skin trouble” and one “not determined”. These adverse events were of mild severity, completely recovered before the end of supplementation and did not lead to discontinuation of supplementation. No serious adverse events were reported.

## Discussion

The results of this observational study suggest that the proprietary supplement containing feverfew, coenzyme Q10 and magnesium could be of interest for migraine prophylaxis. Treatment for 3 months significantly reduced the average number of days with migraine headache in adult patients by more than 3 days, the decrease being progressive over the period of supplementation and statistically significant from first month. The choice of this primary criterion is recommended by the International Headache Society [25]. At baseline, participants presented with the usual characteristics of migraine sufferers consulting GPs (i.e. mainly women in middle age); on average, they had migraine headache for more than 4 days per month with frequent anxiety as co-morbidity and deteriorated quality of life as shown by the QVM questionnaire. After 3 months of supplementation, all parameters evaluated were dramatically improved. With regard to the proportion of patients with a 50% reduction in the number of days with migraine headache, also a classical evaluation criterion for prophylactic treatment of migraine, a significant result was observed from first month and the proportion increased progressively at 2 and 3 months. This observation, which was also made for the primary criterion, suggests that the dietary supplement tested has a rapid onset of action. Such a fast onset was previously reported in the randomised, double-blind, multicentre, placebo-controlled study of Diener et al. [8] with a monopreparation of feverfew (MIG-99, 6.25 mg three times a day); in this study, decrease in the frequency of migraine attacks was significant from the fifth week of treatment. Responder rate in our study was similar to that reported in trials performed with propranolol [26], divalproex sodium [27] and topiramate [28]. These conventional drugs have potential adverse events, sometimes severe in nature. For this reason, patients tend to turn to nutraceuticals and herbal medicines for migraine relief. In our study, the combination of feverfew, coenzyme Q10 and magnesium was well tolerated; only four adverse events of mild severity were reported in four patients. Feverfew, coenzyme Q10 and magnesium monopreparations have not been associated with major safety concerns in previous studies; only mild and transient adverse events, most commonly gastro-intestinal complaints were reported [5, 7, 20]. Now, tolerability and drug safety is a factor influencing compliance and maximization of compliance is one principle of preventive therapy [29]. A meta-analysis has shown that one of six patients discontinue treatment with propranolol, which is the classical first-line drug for migraine prophylaxis [26]. In our study, safety was good.

The first limitation of our study is that it was an observational study and was not randomised, double-blinded and placebo-controlled. Due to the placebo effect, sometimes high in migraine prophylactic treatment [30], the effect of supplementation might be overestimated. Another limitation is the relative modest size of the study population.

Clinical trials performed with feverfew, magnesium and coenzyme Q10 mostly as single agents have shown conflicting results possibly due to different dosages and formulations used [5, 6, 8, 12, 13, 16–19]. To our knowledge, there is no previous report of a study conducted with a combination of feverfew, coenzyme Q10 and magnesium. A randomised clinical trial performed with a combination of feverfew, magnesium and riboflavin failed to show a beneficial effect over control (riboflavin); probably because around one third of patients in the two groups took concomitant prophylactic medication during the study (around 13% in our study) [31]. In a recent randomised double blind placebo-controlled clinical trial performed in 130 patients with a combination of 400 mg riboflavin, 600 mg magnesium and 150 mg coenzyme Q10 [32], the mean number of days with migraine headache per month declined by approximately 2 days after 3 months of treatment; although not statistically significant, this reduction was considered as clinically relevant. In this study, supplementation had a significant beneficial effect on pain and on the impact of headaches on life assessed with the HIT-6 questionnaire.

## Conclusions

This observational study conducted in primary care suggests that the proprietary supplement containing feverfew, coenzyme Q10 and magnesium (Antemig®), at the dose of one tablet per day for up to 3 months, could be effective and well tolerated for the prophylaxis of migraine in adults and deserves further research; the results will have to be confirmed in a randomised placebo-controlled clinical trial. Supplementation with this formulation could be particularly useful in the therapeutic armamentarium to meet the needs of patients suffering from migraine; using it in conjunction with conventional treatments as part of a multidisciplinary treatment plan is likely to result in optimum responses.

## Abbreviations

AAN: American Academy of Neurology
AHS: American Headache Society
EFNS: European Federation of Neurological Societies
GP: General practitioners
HADS: Hospital Anxiety and Depression Scale
ITT: Intention to treat
PP: Per protocol
QVM: Qualité de Vie et Migraine
SD: Standard deviation
